# Uterine Treatment, the Abuse of

**Published:** 1890-08

**Authors:** 


### Selections.

Uterine Treatment, the Abuse of.—Ed. in *The Lancet.*—We have from time to time directed attention to the insignificance of certain conditions of the uterus which have been supposed to hold an important place in the pathology of that organ, and have combated pathological theories which are not only wrong, but which also involve methods of treatment that are often dangerous to life and generally injurious to the health both of body and mind of the patients concerned. It has been a misfortune to women that from the time—now fifty years ago—when attention became directed to the diseases peculiar to the sex, their study has proceeded along very narrow lines, and pathological theories have been formulated upon insufficient and often erroneous data, and made the basis of universal treatment. At one time inflammation of the cervix was the theory generally accepted, and its treatment consisted in the application of caustics to the os uteri once or twice a week for months. After a time stricture of the cervical canal shared the field with inflammation, and this was supposed to be cured by the introduction of bougies at weekly intervals for an indefinite period. Subsequently flexions were discovered, and displacements were soon magnified into an efficient cause of all the ills of women, and upon them was built the superstructure called "the mechanical system of uterine pathology." The treatment by pessaries of all kinds, shapes and substances followed as a matter of necessity, and the pessary required such careful looking after that in many cases it demanded a daily visit. After this the great discovery of conical cervix and pinhole os was made, and chronic inflammation and stricture of the canal rapidly retired from the field. This discovery demanded a cutting operation, and the wearing of a stem for weeks or months. The method proved dangerous, occasionally even fatal; it has, however, been practiced to a large extent, and is still occasionally adopted. Yet later, laceration of the cervix was discovered to be the great cause of suffering in women, and "the greatest surgical triumph of this century" was invented for its cure. This surgical triumph consists in paring the lacerated surfaces and stitching them together. Then massage came to the front. It is true that this mode of treatment is not specially directed to the cure of uterine diseases, but rather to the cure of cases which a few years ago would have been regarded as uterine and would have been treated on one of the plans enumerated above. These are some of the theories which have been held with regard to the diseases of women and the methods employed for their cure. Against these views and methods of treatment the voices of the more thoughtful practitioners in this country have often been raised, but in America "minor gynæcology" of the worst type has always been encouraged, and its practitioners have been regarded as the advance guard of the department, so that we find ourselves pleasantly startled and surprised on reading a paper by a leading American physician, Dr. Goodell, of Philadelphia, on the "Abuse of Uterine Treatment through Mistaken Diagnosis," published in the *Medical News*. We heartily congratulate Dr. Goodell on his emancipation from views, some of which have been proved by laborious researches to be wrong, while others are, on the surface, of such an improbable character that they should be received only on the production of irrefutable proof of their correctness.

It has hitherto been the custom among certain gynæcologists to accept any theory of uterine disease put forward unless it is disproved. No custom has been more disastrous to the scientific progress of this department of medicine, and it should be reversed; no theory, by whomsoever advanced, should be tolerated unless proof of it be forthcoming. Had this rule been observed, uterine pathology would not have been in its present chaotic state, and Dr. Goodell would not have to complain of the abuse of treatment through mistaken diagnosis. Dr. Goodell concludes his paper with the following watchwords as broad helps to diagnosis:

In the first place, "always bear in mind, what another has pithily said, 'that woman has some organs outside the pelvis.' Each neurotic case will have usually a tale of fret or grief, of cark and care, of wear and tear. Scant, or delayed or suppressed menstruation is far more frequently the result of nervous exhaustion than of uterine disease. Anteflexion *per se* is not a pathological condition. It is so when associated with painful menstruation, and only then does it require treatment. An irritable bladder is more often a nerve sympton than a uterine one. In a large number of cases of supposed or actual uterine disease which display marked gastric disturbance, if the tongue be clean, the essential disease will be found to be neurotic; and it must be treated so Almost every supposed uterine case characterized by excess of sensibility and by scantiness of will-power is essentially a neurosis. In the vast majority of causes in which the woman takes to her bed and stays there indefinitely from some supposed uterine lesion, she is bedridden from her brain, and not from her womb. I will go further, and assert this to be the rule, even though the womb itself be displaced, or it is disordered by a disease or by a lesion that is not in itself exacting or dangerous to life. Groin aches and sore ovaries are far more commonly symptoms of nerve exhaustion than of disease of the appendages. Uterine symptoms are not always present in cases of uterine disease. Nor when present and even urgent do they necessarily come from uterine disease, for they may be merely nerve counterfeits of uterine disease."

In the main these conclusions are sound and healthy, but the author shows some hankering after his old views, especially with regard to anteflexion, and a little too much affection for his new love—nerve exhaustion,—a syren which has proved as dangerous as any uterine fad. In spite of these blemishes, we cannot help feeling that the outspokenness of Dr. Goodell will prove an enormous benefit to the study of the diseases of women in America. Already even the effects of this outspokenness are observable. In a discussion on painful menstruation which took place recently in the Obstetrical Society, of Philadelphia, only a small number of the speakers proved themselves to hold orthodox views upon "minor gynæcology." The majority expressed views which were strongly antagonistic to the meddlesome local treatment which has been in favor so long a time. This is highly satisfactory. We shall accordingly look forward with pleasure to sound scientific and pathological work from the obstetricians of America, instead of the "inventions" in pessaries, instruments, and minor operations for conditions of which so little is known.—*The Archives of Gynæcology, Obstetrics and Pædiatrics, June, 1890.*

